# From hospital to primary care: the importance of coordinated care in peripheral arterial disease: a commentary

**DOI:** 10.1177/17449871251347159

**Published:** 2025-12-02

**Authors:** Pernilla Garmy

**Affiliations:** Professor, Faculty of Health Sciences, Kristianstad University, Sweden

Commentary to: The transitional care program from hospital to primary care setting in peripheralarterial disease of lower limbs after surgery: a scoping review (Mozzarelli et al., 2025).

Up to 10% of the global population may suffer from peripheral arterial disease (PAD), a condition characterised by reduced blood flow to the legs due to atherosclerosis. This leads to considerable individual suffering and places a significant burden on healthcare systems. Improving care pathways for these patients offers the dual benefit of reducing personal distress and lowering societal healthcare costs.

In some cases, PAD requires vascular surgical interventions. This scoping review by Mozzarelli et al. (2025) explored the transition from hospital to primary care for patients with PAD and examined the role of the Transitional Care Program (TCP) in this process—a model previously described in the literature (Storm et al., 2014). In many countries, the majority of care is delivered in the primary care setting. There is a clear trend towards shorter hospital stays, followed by continued care at home or in residential facilities. This shift places significant demands on effective transitions and strong interprofessional communication. Self-care and lifestyle management are also essential, with smoking and poorly controlled diabetes recognised as key risk factors for PAD.

The TCP model aims to coordinate the transition from hospital to primary care by involving a specifically designated nurse – a Care Transition Nurse (CTN) – as a central point of contact. In their review, Mozzarelli et al. screened 888 papers, of which 6 papers met the inclusion criteria. The findings suggested that TCP contributed to a reduction in hospital readmissions within 30 days and to improved quality of life, particularly in healthcare structures that included a CTN.

No significant differences were observed in complications such as unplanned surgeries, major amputations or mortality. The authors concluded that TCP enhanced patient outcomes when it incorporated education and telemedicine within a multiprofessional care framework, with the CTN serving as a key liaison for patients, caregivers, healthcare teams and organisations.

Transitioning between levels of care remains a recognised challenge in healthcare systems. Although professionals are often highly skilled within their respective domains, coordination across services continues to be a vulnerable link. Here, TCP offers a structured and holistic approach to bridging these gaps.

This scoping review addresses an important knowledge need (Peters et al., 2024). By including both scientific and grey literature, the authors provided a comprehensive overview of the current state of evidence. The findings reinforce the idea that well-coordinated care transitions can improve both clinical outcomes and patient experiences – particularly relevant for patients with PAD, who are often multimorbid, frail and in need of tailored post-surgical support.

TCP represents a promising model within integrated care. By leveraging technologies such as telemedicine, it can support healthcare professionals, patients and their families during vulnerable periods. By fostering continuity of care and reducing preventable readmissions, TCP offers a compelling model for improving outcomes in vulnerable surgical populations, including those with PAD.
